# Dextran sodium sulfate‐induced colitis in male BALB/c mice leads to albuminuria and increased markers of inflammation and tissue damage in the kidney

**DOI:** 10.14814/phy2.70161

**Published:** 2025-02-28

**Authors:** Hanne Salmenkari, Krishna Adeshara, Anniina Pirttiniemi, Jere Lindén, Sanna Lehtonen, Niina Sandholm, Per‐Henrik Groop, Markku Lehto

**Affiliations:** ^1^ Folkhälsan Research Center Helsinki Finland; ^2^ Research Program for Clinical and Molecular Metabolism University of Helsinki Helsinki Finland; ^3^ Department of Nephrology University of Helsinki and Helsinki University Hospital Helsinki Finland; ^4^ Department of Veterinary Biosciences University of Helsinki Helsinki Finland; ^5^ Finnish Centre for Laboratory Animal Pathology HiLIFE, University of Helsinki Helsinki Finland; ^6^ Department of Pathology University of Helsinki Helsinki Finland; ^7^ Department of Diabetes Central Clinical School, Monash University Melbourne Australia; ^8^ Baker Heart and Diabetes Institute Melbourne Victoria Australia

**Keywords:** dextran sodium sulfate, extraintestinal manifestations, gut‐kidney axis, kidneycolitis

## Abstract

Inflammatory molecules originating from an inflamed gut can promote systemic inflammation. We studied how acute intestinal injury affects the kidneys and the kallikrein‐kinin system in mice with dextran sodium sulfate (DSS)‐induced colitis. Seven‐week‐old male BALB/c mice were treated with 5% DSS for 7 days and either sacrificed immediately (DSS7, *n* = 6) or given fresh water for 4 more days (DSS11, *n* = 6). Untreated mice (n = 6) served as controls. Colitis and kidney damage was assessed using histochemical and immunohistochemical staining, ELISA, and RT‐qPCR. Markers of kidney injury correlated with markers of colitis. Colitis increased albuminuria, reduced kidney weight, and induced transcription of *lipocalin 2*, *kidney injury molecule‐1*, and *interleukin‐1beta*, as well as increased immunostaining signal of c‐Jun and NF‐κB p65 in the kidneys. Colitis caused strong induction of colonic *kininogen 2* transcription and bradykinin receptor B1‐positive cells in the disrupted mucosa. In the kidney, colitis induced localization of tubular bradykinin receptor B2 to the nuclear envelope and increased *kininogen 2* transcription. Disruption of the intestinal barrier by DSS promotes markers of kidney injury and inflammation, and the degree of kidney injury correlates with the severity of colitis. Colitis is associated with increased expression of kallikrein‐kinin components in both the colon and kidneys.

## 1 INTRODUCTION

People with inflammatory bowel disease (IBD) have an increased risk to develop inflammatory kidney injury, and intestinal disease activity correlates with the appearance of kidney manifestations (Corica & Romano, 2016). Microbial toxins and proinflammatory compounds originating from the gut may lead to extraintestinal pathologies ranging from low‐grade inflammation to organ damage such as kidney disease. Acute kidney injury (AKI) and chronic kidney disease (CKD) lead to a decline in kidney function, including disturbances in the removal of metabolic waste products, regulation of blood pressure, and electrolyte balance either reversibly or permanently. The main causes of AKI are infections, toxins, and hypoxia, in which inflammation in the kidneys play an important role (Akcay et al., 2009). Although diabetes and hypertension are the main risk factors for CKD, AKI itself may also eventually lead to the development of chronic kidney disease. Both IBD and diabetes increase the risk of various infections (Benfield et al., 2007; Thomsen et al., 2011), of which particularly gastroenteritis is associated with an increased risk of AKI (Mansfield et al., 2018). Bacterial infections and endotoxemia are associated with the progression of diabetic complications such as coronary heart disease, retinopathy, and diabetic kidney disease (Nymark et al., 2009; Simonsen et al., 2020, 2021). Gut dysbiosis (Bielka et al., 2022; Santana et al., 2022), increased intestinal permeability (Carratu et al., 1999; Horton et al., 2014), and higher serum bacterial lipopolysaccharide (LPS) concentrations (Cox et al., 2017; Gardiner et al., 1995; Gomes et al., 2017; Nymark et al., 2009) are frequently present in people with IBD or diabetes. Based on this background, we hypothesized that gut‐related disorders may significantly contribute to kidney pathologies.

According to our recent genome‐wide association study in the Finnish population, the serum LPS activity levels were significantly associated with the genes involved in the regulation of contact activation pathway and the kallikrein‐kinin system (KKS) (e.g. FXI, FXII, KLKB1, KNG1, and C1‐INH) (Leskela et al., 2021). The KKS leads to production of kinin peptides and activation of bradykinin receptors B1 (BK1) and B2 (BK2), which control inflammation, vasodilation, and vascular permeability. The precursors to the kinin peptides are splice variants of the *kininogen* (*Kng*) gene product and are cleaved to bradykinin and kallidin by plasma and tissue kallikreins, respectively. They have high affinity to the constitutively expressed BK2, and further enzymatic dearginization of these peptides increases their affinity to the inflammation‐induced BK1 (Bekassy et al., 2022). Mice harbor two kininogen‐encoding genes in their genome, of which *Kng1* majorly contributes to plasma kininogen, whereas the product of *Kng2* is not secreted (Yang et al., 2017). Involvement of the intestinal KKS has been found in experimental colitis and IBD (Stadnicki et al., 2003; Wang et al., 2018). Furthermore, deficiency in complement 1 inhibitor (C1‐INH), the main endogenous inhibitor of plasma kallikrein, has recently been linked to increased risk of kidney disease (Christiansen et al., 2023).

The primary aim of the present study was to investigate the causal relationships between the disrupted gut barrier function and kidney injury in BALB/c mice. We employed the mouse dextran sodium sulfate (DSS) model of colitis to induce gut inflammation and intestinal barrier damage. The relationship between gut and kidney associated injuries, as well as the role of KKS pathway components in related pathogenic processes, were evaluated with tissue‐specific biomarkers and histological examination.

## 2 METHODS

### 2.1 Experimental setup and induction of colitis

The animal experiments were authorized by the Finnish Project Authorization Board under the permit ESAVI/6504/2020. Eighteen male BALB/c mice from Scanbur (Sollentuna, Sweden) were used in the experiment. The mice were housed in pairs in conventional cages with environmental enrichment and kept in a Scantainer rack system under 12:12‐h light–dark cycle. The mice were allowed to acclimatize for at least 9 days before the start of the experiment.

To induce colitis, twelve seven‐week‐old BALB/c male mice were given 5% (w/vol) 40 kDa DSS (DB001, TdB Labs, Uppsala, Sweden) for a total of 7 days in the drinking water. The colitis mice were randomly divided into two groups, six mice were sacrificed after 7 days of DSS administration (DSS7 group) and six were allocated to receive fresh water for 4 days after removal of DSS (DSS11 group). The mice in the DSS11 group were sacrificed either on day 11 or when they met humane endpoints, in which case the mice were sacrificed and samples collected in the same manner as other mice and analyzed together with the rest of the DSS11 group (three mice sacrificed on day 10). Six healthy untreated mice were used as controls. Standard chow (#Teklad 2916, Inotiv) and drinking fluid (5% DSS or water) were available ad libitum. Spot urine and fecal samples were collected on the day of the sacrifice. The mice were weighed daily and monitored for diarrhea and rectal bleeding.

At the end of the study, mice were sacrificed by exsanguination under isoflurane anesthesia. Blood was collected in gel filter tubes, and sera were separated after 30 min incubation at RT by centrifuging for 3 min at 10000*g*. Kidneys were excised and weighed together. The kidney capsule was removed, and samples were taken for paraffin embedding and RNA extraction. The colon was excised, and its length measured. For macroscopic scoring of the colitis, the colons were opened longitudinally, and the stool consistency, blood, and edema were scored. The scoring was done as described previously (Salmenkari et al., 2018). The stool consistency was evaluated on a scale from 0 to 4 (0 = normal, 1 = moist, 2 = loose, 3 = liquid, 4 = none), bleeding on a scale from 0 to 3 (0 = none, 1 = streaks, 2 = clearly visible, 3 = heavy bleeding), after the colons were cleaned of intestinal contents edema was scored on a scale of 0 to 3 (0 = normal, 1 = mild, 2 = moderate and 3 = marked). The scores were combined as a total macroscopic score (scale 0–10). Samples from colons were taken for paraffin embedding and RNA extraction. Kidney and colon tissues were fixed in 10% neutral‐buffered formalin for 24 h at RT, rinsed with phosphate‐buffered saline (PBS), and stored in 70% ethanol at +4°C until paraffin embedding. The embedding of paraffin blocks was done at the Tissue Preparation and Histochemistry Unit (TPHU) Meilahti and the sectioning at FCLAP at the University of Helsinki, Finland. The samples for RNA extraction were flash frozen in liquid nitrogen and kept at −80°C until processed.

### 2.2 Histopathological evaluation of colitis and kidney injury

Paraffin‐embedded longitudinal colon samples and kidney samples were cut to 4 μm thick sections and stained with hematoxylin and eosin (H&E) and periodic acid‐Schiff (PAS), respectively, at FCLAP. Histopathological scores of the colons were assessed by a modified scoring from (Erben et al., 2014), where inflammatory infiltrate, and erosions were each scored on a range from 0 to 3. An additional multiplier to the infiltrate score was given based on the extent of the infiltrate (0.5x if <10%, 1x if 10–25%, 1.5 if 24–50%, and 2x if >50% of the length of the section) to better account for the patchy nature of the DSS colitis. Kidney sections were visually evaluated for vacuolization, pyknosis, tubular dilation, proximal tubule brush border anomalies, and glycolipid deposition.

### 2.3 Immunofluorescence staining

Protein expression and localization was evaluated by immunofluorescence labeling of formalin‐fixed paraffin‐embedded colon and kidney samples sectioned to 4 μm thickness. Each staining was performed simultaneously for all 18 mice. Deparaffinization and rehydration were performed using a standard xylene and ethanol series. Antigen retrieval was done by boiling the slides in microwave in 10 mM Tris–HCl—1 mM EDTA—0.05% Tween 20, pH 9 for 20 min. The slides were blocked in 5% fetal bovine serum (FBS)—0.1% Triton X‐100 in PBS for 1 h at RT. The tissue slides were incubated with primary antibodies diluted in 1% FBS in PBS overnight at +4°C. Slides were treated with secondary antibodies and Hoechst 33258 (H3569, Thermo Fisher Scientific) diluted in 1% FBS in PBS for 1 h. The kidney slides were incubated in TrueView autofluorescence quenching solution (SP‐8400, Vector Laboratories) for 5 min and mounted with VectaShield Vibrance mounting medium (H‐1700, Vector Laboratories). Colon samples were mounted with Mowiol‐Dabco mounting medium (81,381 and D27802, Merck). The antibodies and their dilutions used in immunofluorescence staining were; the primary antibodies against mouse nephrin 1:400 (GP‐N2, Progen Biotechnik), NF‐κB 1:400 (D14E12, Cell Signaling Technology), c‐Jun 1:200 (60A8, Cell Signaling Technology), CD45 1:200 (70,257, Cell Signaling Technology), kallistatin/SERPINA4 (PA5‐96636, Thermo Fisher), bradykinin B1 receptor (BK1) 1:200 (BS‐8675R, Bioss), and bradykinin B2 receptor (BK2) 1:200 (NBP1‐46328, Novus Biologicals), and secondary Alexa Fluor labeled antibodies anti‐Rabbit‐AF594 1:750 (A‐21207, Thermo Fisher Scientific) and anti‐Guinea pig‐AF488 1:750 (A‐11073, Thermo Fisher Scientific). The slides were imaged using an Axio Imager M2 microscope, equipped with Axiocam 503 (Carl Zeiss Microscopy GmbH, Oberkochen, Germany). Images were edited using Zen 3.1 Imaging software and all adjustments were done identically to the whole staining set.

### 2.4 Quantitative reverse transcription polymerase chain reaction

Transcription of target genes were analyzed using quantitative reverse transcription PCR (RT‐qPCR). RNA from colon and kidney samples were extracted using Nucleospin RNA (#740955, Macherey‐Nagel). One μg of RNA was reverse transcribed to cDNA using iScript cDNA Synthesis Kit (#1708891, Bio‐Rad). RT‐qPCR was carried out using iTaq Universal SYBR Green Supermix (#1725121, Bio‐Rad) in CFX384 Touch Real‐Time PCR Detection System (Bio‐Rad, Hercules, CA). The samples were denatured in 95°C for 30 s, followed by 40 cycles alternating between denaturation at 95°C for 5 s and amplification at 60°C for 30 s. Primer sequences are presented in Table 1. Target mRNA expression was normalized to the geometric mean of *Eef2*, *Rplp0* and *S18* expression. Individual fold changes were calculated against the geometric mean of the target gene expression in the healthy mice.

**TABLE 1:** Primer sequences.

Target	Forward primer	Reverse primer	Reference
*Eukaryotic translation elongation factor 2, Eef2*	5′‐TGTCAGTCATCGCCCATGTG‐3′	5′‐CATCCTTGCGAGTGTCAGTGA‐3′	Eissa et al. (2016)
*Ribosomal protein lateral stalk subunit P0, Rplp0*	5′‐CATCCTTGCGAGTGTCAGTGA‐3′	5′‐GGTACCCGATCTGCAGACA‐3′	Salmenkari et al. (2018)
*Ribosomal protein S18,18S*	5′‐AACGAACGAGACTCTGGCAT‐3′	5′‐ACGCCACTTGTCCCTCTAAG‐3′	Salmenkari et al. (2017)
*Interleukin 1 beta, Il‐1β*	5′‐CTCCAGCCAAGCTTCCTTGT‐3′	5′‐TCATCACTGTCAAAAGGTGGCA‐3′	Salmenkari et al. (2018)
*Kidney‐injury molecule‐1, Kim‐1*	5′‐TTGCCTTCCGTGTCTCTAAG‐3	5′‐AGATGTTGTCTTCAGCTCGG‐3'	Nakano et al. (2020)
*Tumor necrosis factor, Tnfa*	5′‐TGGCACCACTAGTTGGTTGTCT‐3′	5′‐AGCCTGTAGCCCACGTCGTA‐3′	Nakano et al. (2020)
*Lipocalin 2, Lcn2*	5′‐CCACCACGGACTACAACCAG‐3′	5′‐AGCTCCTTGGTTCTTCCATACAG‐3′	
*Interleukin 6, Il6*	5′‐ATCGTGGAAATGAGAAAAGAGTTGT‐3′	5′‐CTGCAAGTGCATCATCGTTGT‐3′	
*Transforming growth factor, beta 1, Tgfb1*	5′‐ACGTCAGACATTCGGGAAGC‐3′	5′‐GTTCCACATGTTGCTCCACAC‐3′	
*Interferon‐induced protein with tetratricopeptide repeats 2, Ifit2*	5′‐ACTGGAGAGCAATCTGCGAC‐3′	5′‐CAGTATGTTGCACATGGTGGC‐3′	
*Nephrin, Nphs1*	5′‐AGAAGCTCCACGGTTAGCAC‐3′	5′‐CCTGTGAAGGCTTGGCGATA‐3′	
*Podocalyxin‐like, Podxl*	5′‐TGCAACAGTCTATGGCGTCT‐3′	5′‐GGAGGTTCACAGTTTAGCTGGT‐3′	
*Kininogen 2, Kng2*	5′‐AGCTGTGACCTTCATCCAGGA‐3′	5′‐TGACCAAGCACCTCCTTCAG‐3′	
*Acyloxyacyl hydrolase, Aoah*	5′‐CAGCTACCTGCCTGAAAAGC‐3′	5′‐GCATAGCACACCACATCAGC‐3′	
*Alkaline phosphatase, intestinal, Alpi*	5′‐ACCGAAGCTCAGAGTGTTGAT‐3′	5′‐GCAAATATGGCCACGTCCTC‐3′	
*Angiotensin‐converting enzyme, Ace*	5′‐GCTGGAGGGTCTTTGATGGA‐3′	5′‐AGTCACCTTGGGATCTTGGC‐3′	

### 2.5 Fecal samples

Soluble proteins were extracted from fecal samples by vortexing in 10x volume (w/vol) of extraction buffer (10 mM Tris–HCl, 1 mM MgCl2, 0.1 mM ZnCl2, pH 8) containing cOmplete™, EDTA‐free Protease Inhibitor Cocktail (#04693159001, Roche, Mannheim, Germany) for 10 min. The supernatants were centrifuged and moved to clean tubes twice, first for 5 min at 5000 *g*, and then for 15 min at 16000 *g* at +4°C.

### 2.6 Other assays

Fecal calprotectin was assayed with Mouse S100A8/S100A9 Heterodimer DuoSet ELISA (#DY8596‐05, R&D Systems, Minneapolis, MN), and the values were normalized to the total protein concentration of the fecal sample measured by using the Pierce™ BCA Protein Assay Kit (#23227, Thermo Scientific, Waltham, MA, USA). Urinary and fecal albumin concentrations were measured using Mouse Albumin ELISA Kit (#E99‐134, Bethyl Laboratories, Waltham, MA, USA). Urinary albumin levels were normalized to urinary creatinine levels which were assayed using Invitrogen™ Creatinine Urinary Detection Kit (#EIACUN, Thermo Fisher Scientific). Serum was assayed for circulating cytokine concentrations of IFNγ, IL‐1β, IL‐6, and TNFα using a multiplex Q‐Plex Mouse Cytokine Panel 2 (#115549MS, Quansys Biosciences, Logan, UT, USA).

### 2.7 Statistical analysis

Data in bar graphs are presented as means ± standard deviations. Gene expression data are presented as geometric means and geometric standard deviations (equal to arithmetic means ± standard deviations of log_10_ transformed expression values). Graphs show *p*‐values under 0.05 unless otherwise indicated. Nonparametric data and RT‐qPCR results were analyzed using Kruskal‐Wallis test, followed by Pairwise Wilcoxon Rank Sum Test with Holm correction for multiple comparisons in R version 4.2.2. Other analyses were conducted with GraphPad Prism software version 8 (GraphPad Software, San Diego, CA). Weight development was compared against healthy controls using mixed‐effects analysis and adjusted for multiple comparisons using Sidak's test. Parametric end‐point data were analyzed using ANOVA and Holm‐Sidak post hoc test. Spearman correlations between parameters were calculated using data from all groups.

## 3 RESULTS

### 3.1 DSS administration leads to progressive colitis

**FIGURE 1:**
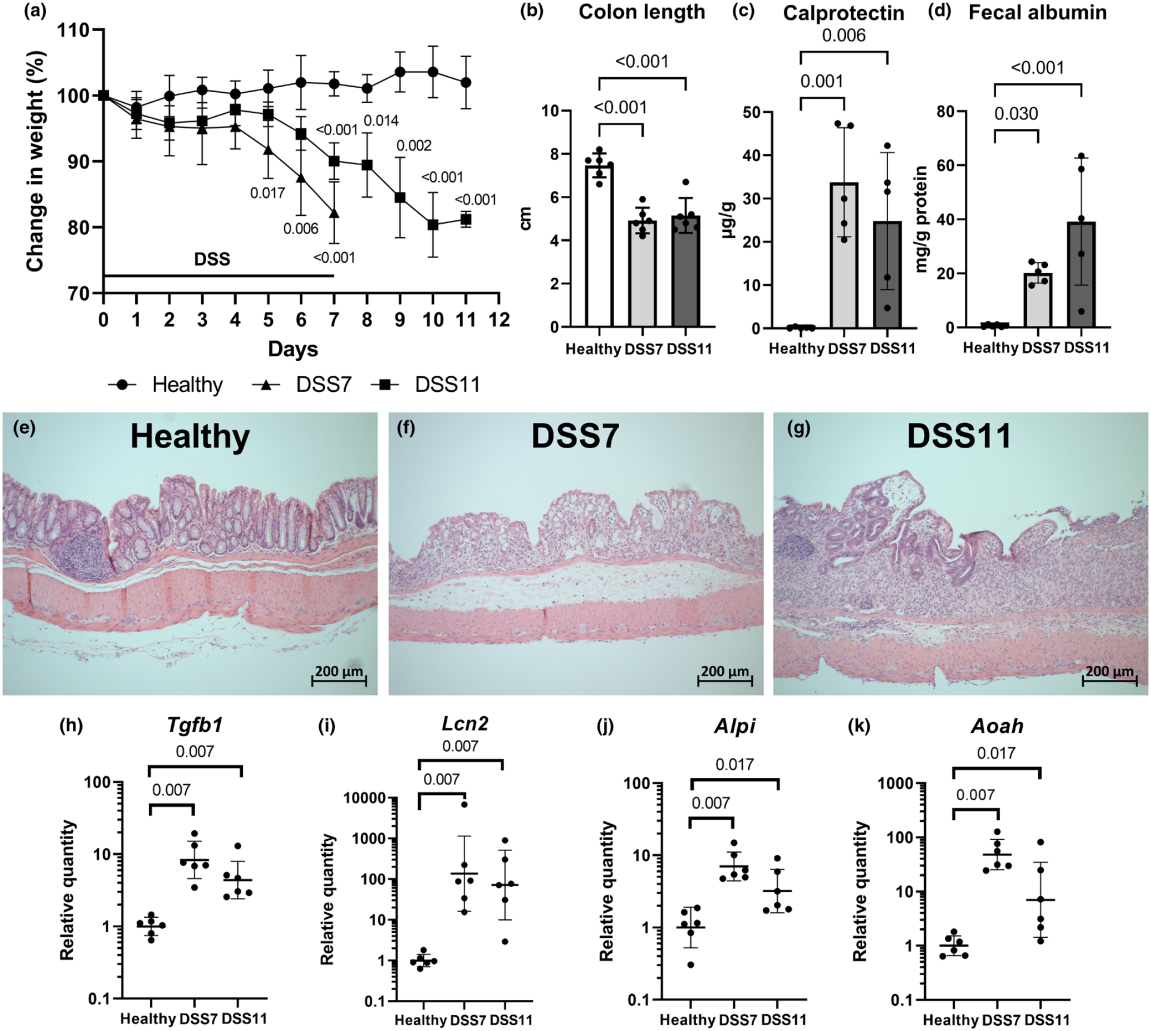
Colitis progression. The DSS treatments induced a prominent colitis at both timepoints based on weight loss (*p*‐values <0.02 shown) (a), shortened colon lengths (b), increase in fecal calprotectin (c), fecal albumin levels (d), and histological damage (e–g). Colonic gene expression of *Tgfb1*, *Lcn2*, *Alpi*, and *Aoah* analyzed using RT‐qPCR (h–k). Data in weight and bar charts are presented as means ± standard deviations. RT‐qPCR data are presented with geometric means ± geometric standard deviations. *Alpi* = intestinal alkaline phosphatase, *Aoah* = acyloxyacyl hydrolase, DSS = Dextran sodium sulfate, *Lcn2* = lipocalin 2, *Tgfb1* = transforming growth factor beta 1.

To disrupt the gut mucosal barrier and induce translocation of bacteria and bacterial products, the mice were treated with DSS for 7 days and either sacrificed right away or allocated to be given water for four more days to allow the colitis to develop further. The mice in both the DSS7 and the DSS11 colitis groups displayed prominent signs of colitis, including diarrhea, rectal bleeding, and weight loss (Table 2, Figure 1a). After 7 days, the mice in the DSS7 group had lost an average of 17.8% of their body weight. In the DSS11 group, the weights continued to decline after the removal of DSS and three of the mice were sacrificed after 3 days of water administration and three after 4 days. The colon lengths were shortened in both colitis groups, and the mice exhibited loose colonic content and prominent edema (Figure 1b, Table 2). The mice in the DSS7 group presented with bloody colonic content, and the bleeding was slightly reduced in the DSS11 mice during the days after DSS removal.

**TABLE 2:** Colitis parameters. Combined macroscopic scores, consisting of stool consistency, colonic bleeding and edema, and histopathology scores were increased by colitis.

Group	Healthy	DSS7	DSS11
Stool consistency	0	3 (1)*	3 (1)*
Colonic blood	0	2 (2)*	0 (2)
Colonic edema	0	3 (1)*	3 (0) *
Macroscopic score	0	7 (3)*	7 (1)*
Histopathology score	0	5 (2)*	9 (7)*

*Note*: *n* = 6 in each group. Numbers expressed as median (interquartile range).

Abbreviation: DSS, Dextran sodium sulfate.

* *p* < 0.05 compared to healthy control.

Fecal calprotectin levels, a marker of neutrophilic inflammation, was strongly induced by DSS colitis at both timepoints (Figure 1c). Similarly, fecal albumin, reflecting increased intestinal bleeding and permeability, was increased at both timepoints (Figure 1d). In histopathological evaluation of the colon (Table 2, Figure 1e–g), the mice in the DSS7 group exhibited prominent inflammation and mucosal damage manifested as disruption of crypt morphology, loss of goblet cells, and extensive erosion of mucosal surface epithelium (Figure 1f). The DSS11 group mice displayed epithelial erosions, dense inflammatory infiltrates in the lamina propria, crypt abscesses, thickening of the mucosa, and a variable degree of crypt loss, regeneration, and hyperplasia (Figure 1g). Colonic mRNA expression of *Tgfb1* and the tissue damage marker *Lcn2* were upregulated in both DSS groups (Figure 1h,i). In addition, two genes encoding LPS‐detoxifying enzymes, *Alpi* encoding intestinal alkaline phosphatase, and *Aoah* encoding acyloxyacyl hydrolase, were prominently induced by colitis (Figure 1j,k).

**FIGURE 2:**
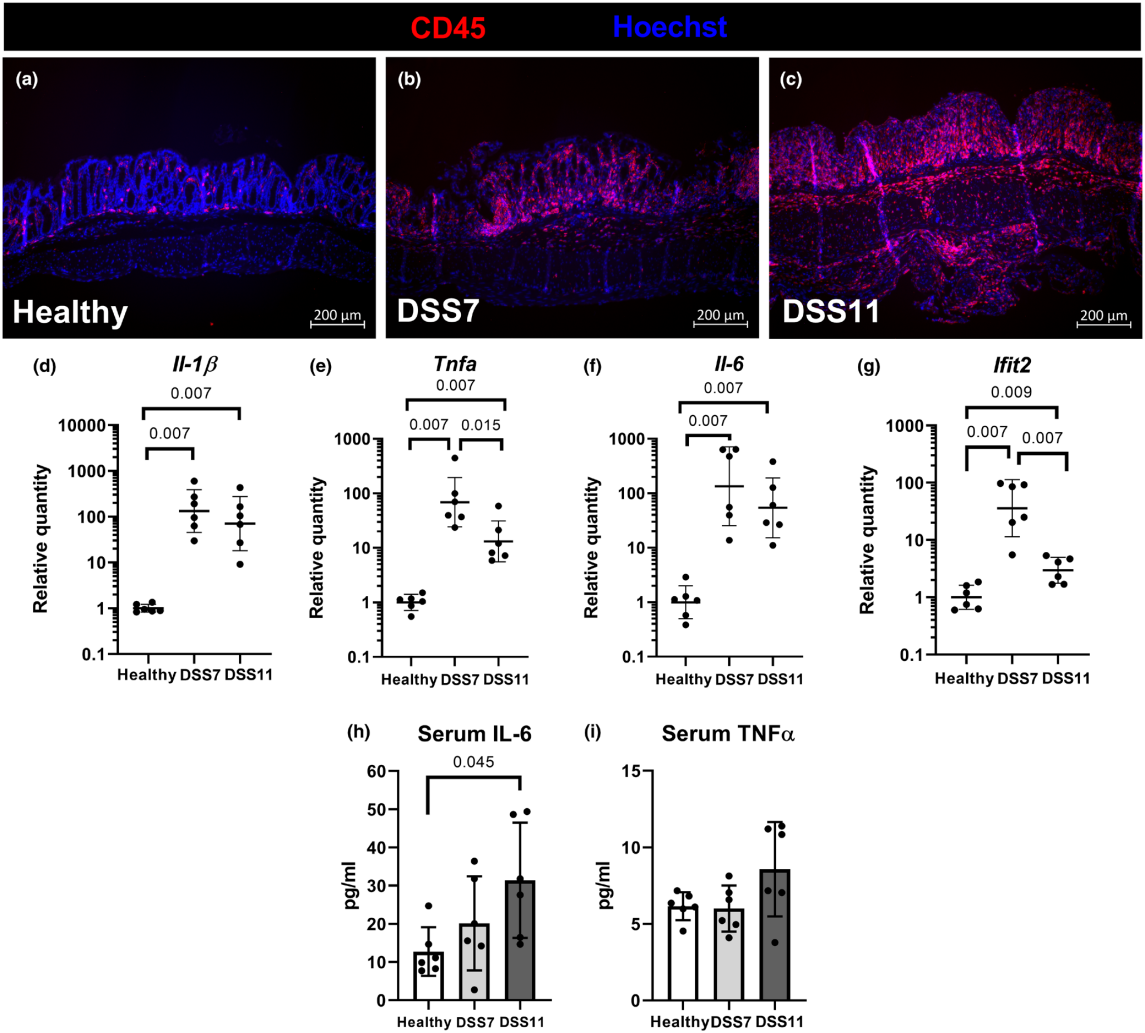
Colitis is associated with systemic inflammation. Leukocytes visualized by immunostaining against CD45 in colon during colitis progression (a–c). Colitis was accompanied by a marked induction of the proinflammatory cytokines and markers *Il‐1β*, *Tnfa*, *Il‐6*, and *Ifit2* (d–g). Systemic inflammation was indicated by an increase in serum IL‐6 concentrations after 11 days (h). Serum TNFα (i). RT‐qPCR data are presented with geometric means ± geometric standard deviations. Data in bar charts are presented as means ± standard deviations. DSS = Dextran sodium sulfate, *Ifit2 =* interferon‐induced protein with tetratricopeptide repeats 2, IL = interleukin, *Tnfa* = tumor necrosis factor alpha.

Leukocyte staining with the CD45 antibody (Figure 2a–c) revealed mucosal and submucosal inflammatory infiltrate in the DSS7 group mice (Figure 2b), which over time advanced to dense inflammatory infiltrate in the mucosa and transmural inflammation reaching the muscularis externa in the DSS11 group (Figure 2c). Overall, the mice developed moderate colitis during the 7 days of DSS treatment, which progressed to prominent histopathological damage during the days after removal of DSS. This was accompanied by a prominent increase in colonic mRNA of the proinflammatory cytokines *Il‐1β*, *Tnfa*, and *Il‐6* as well as the interferon‐stimulated gene, *Ifit2*, in mice with colitis compared to the healthy controls (Figure 2d–g). Systemic inflammation was evaluated by measuring proinflammatory cytokine concentrations in the sera of the mice at the end of the experiment. Of the cytokines included in the panel, serum IL‐6 concentrations were elevated in the DSS11 group, indicating active systemic inflammation (Figure 2h). The increase in serum TNFα was not statistically significant (Figure 2i), and the IL‐1β and IFNγ concentrations remained below the detection limit of the kit (data not shown).

### 3.2 Colitis induces markers of kidney injury and inflammation

**FIGURE 3:**
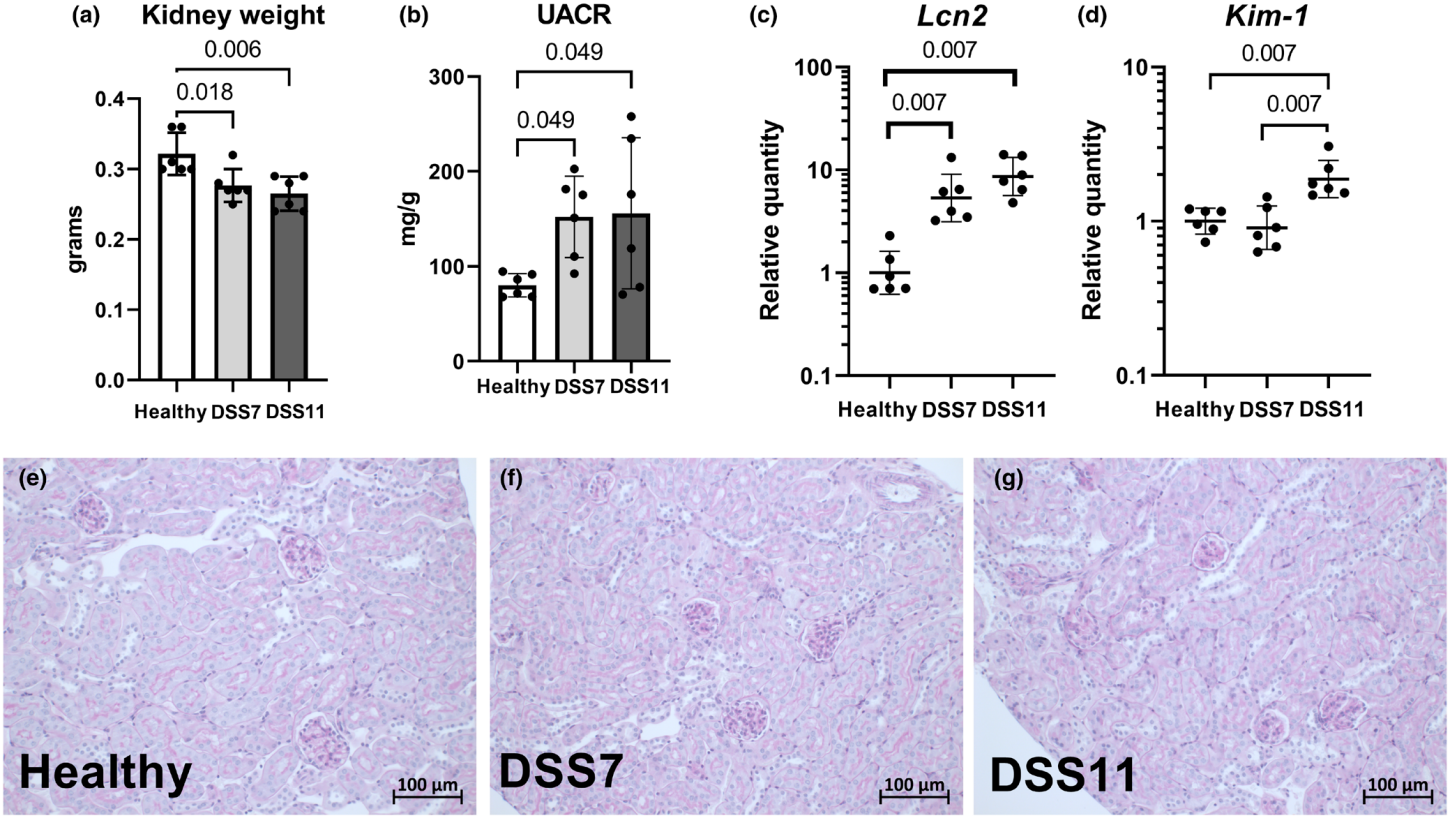
Markers of kidney injury are elevated by DSS colitis. Kidney weight (a), urinary albumin‐to‐creatinine ratios (b), kidney expression of *Lcn2* and *Kim‐1* analyzed with RT‐qPCR (c–d), PAS stainings of mouse kidneys (e–g). Data in bar charts are presented as means ± standard deviations. RT‐qPCR data are presented with geometric means ± geometric standard deviations. DSS = Dextran sodium sulfate, *Kim‐1* = kidney‐injury molecule‐1, *Lcn2* = lipocalin 2, PAS = periodic acid‐Schiff, RT‐qPCR = quantitative reverse transcription polymerase chain reaction, UACR = urinary albumin‐to‐creatinine ratio.

We evaluated several markers of kidney injury and inflammation at the two DSS colitis timepoints and found several parameters relevant in human kidney disease to be altered by colitis. DSS treatment led to a reduction in kidney weights in the colitis groups (Figure 3a), possibly due to dehydration. Albuminuria is one of the most important markers of kidney damage. Urinary albumin‐to‐creatinine ratio (UACR) was increased at both timepoints at the end of the experiment (Figure 3b). Kidney transcription of *Lcn2*, a highly sensitive tissue damage marker, was strongly elevated by colitis (Figure 3c). The expression of another kidney injury marker, *Kim‐1*, was only increased at the later timepoint in the DSS11 mice (Figure 3d). However, in histology, the DSS colitis mice kidneys showed no overt glomerular or tubular histopathological changes in the PAS‐stained sections (Figure 3d–f).

**FIGURE 4:**
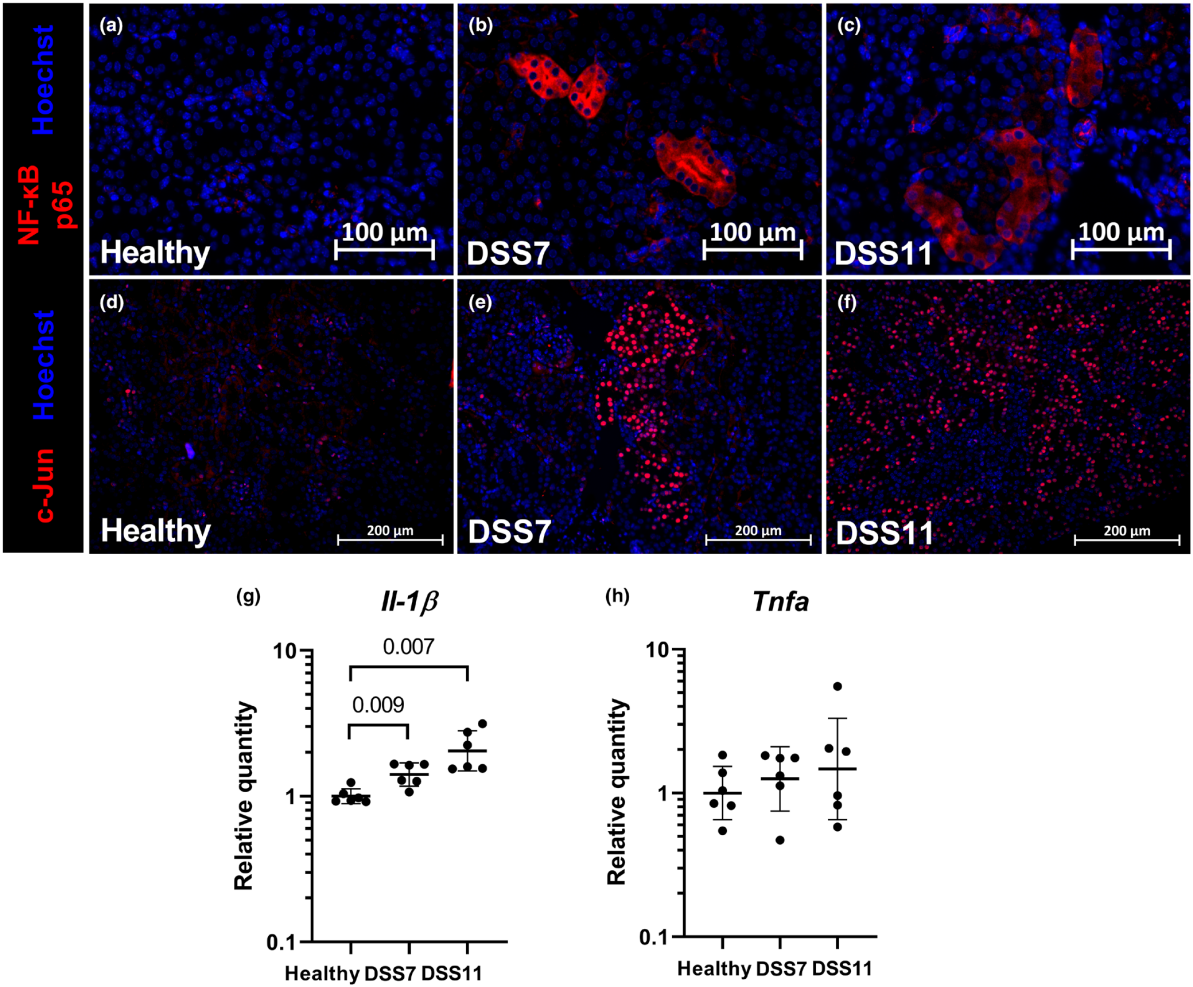
Immunofluorescence staining reveals increased inflammation in the kidneys of colitis mice. Immunostaining of NF‐κB (a–c) showed positive staining pattern in some individual tubules in DSS7 and DSS11 colitis groups. c‐Jun expression (d–f) was increased and localized to nuclei either segmentally or ubiquitously in DSS7 and DSS11 groups, whereas in healthy mice, strong c‐Jun staining was observed only in very few individual tubules (d–e). RT‐qPCR analysis of *Il‐1β* (g) and *Tnfa* (h). Data are presented with geometric means ± geometric standard deviations. DSS = Dextran sodium sulfate, *Il‐1β* = interleukin 1 beta, NF‐κB = nuclear factor‐kappa B, *Tnfa* = tumor necrosis factor alpha.

Although kidney histology in colitis mice seemed normal, immunofluorescence staining revealed changes in inflammatory markers. Immunofluorescence NF‐κB staining was low in healthy mice (Figure 4a), while in the colitis groups some individual tubules showed strong positive cytoplasmic staining (Figure 4b,c). In the healthy kidneys, sparse individual c‐Jun transcription factor‐positive nuclei scattered in some of the tubules (Figure 4d), and very rarely, there was clustering of a stronger positive signal in individual tubules. On the contrary, the colitis mice displayed increased staining and nuclear localization of the c‐Jun (Figure 4e,f), and the extent of clustering followed the severity of the colitis from segmental clustering in the DSS7 group (Figure 4e) to uniform nuclear staining at the later timepoint in the DSS11 mice (Figure 4f). Of the proinflammatory cytokines, kidney *Il‐1β* transcription was increased in both DSS groups, but *Tnfa* transcription did not differ from healthy controls (Figure 4g,h).

**FIGURE 5:**
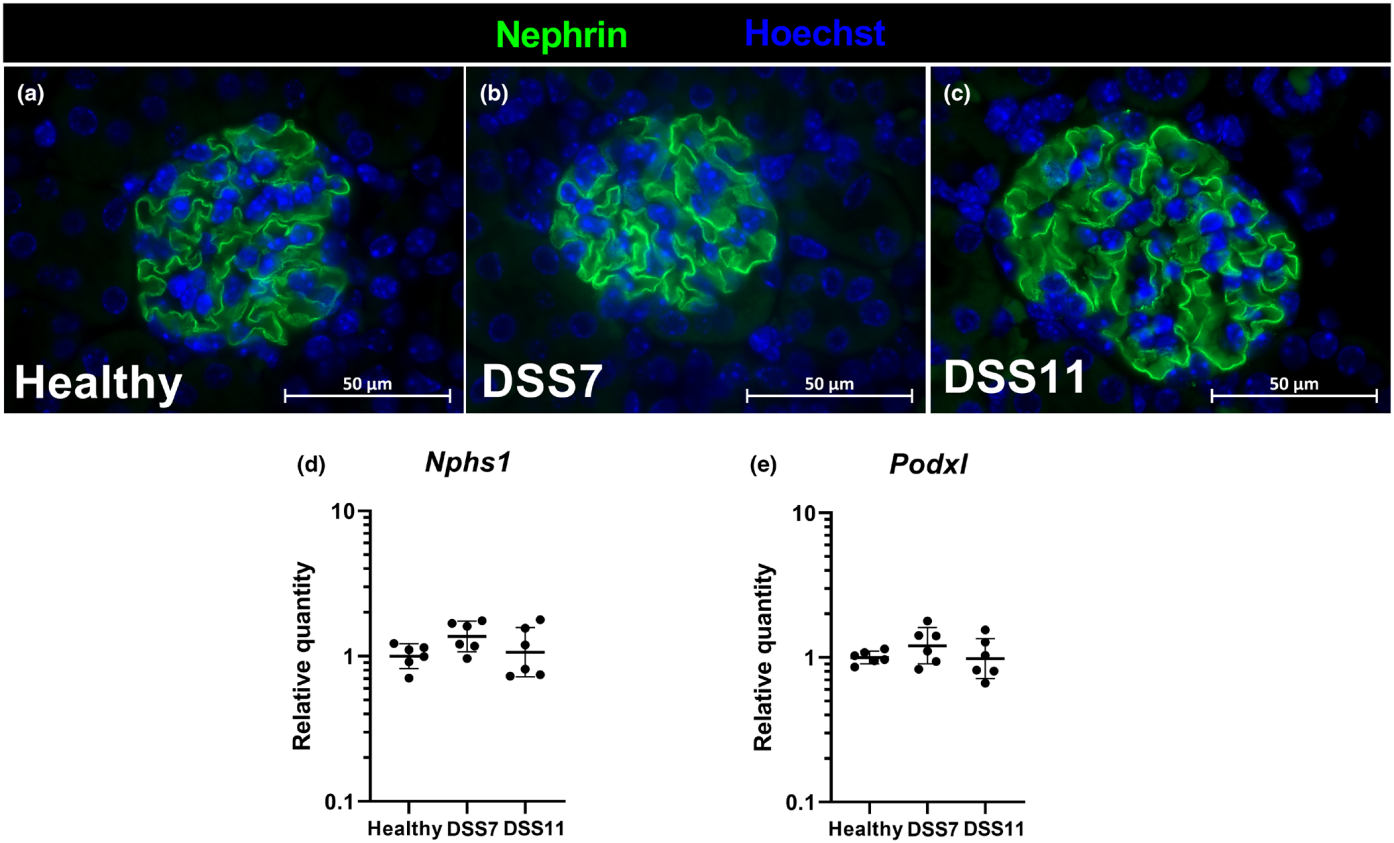
Podocyte proteins. Immunofluorescence staining of the podocyte protein nephrin in mouse glomeruli (a–c). mRNA expression of *Nphs1* (d), and *Podxl* expression (e) measured with RT‐qPCR. Data are presented with geometric means ± geometric standard deviations. DSS = Dextran sodium sulfate, *Nphs1* = nephrin, *Podxl* = podocalyxin‐like.

We investigated the expression of the podocyte proteins nephrin and podocalyxin using immunofluorescence staining and RT‐qPCR. The DSS treatment group glomeruli displayed normal appearance and we found no change in the expression pattern of the podocyte protein nephrin in immunohistochemical evaluation. Similarly, no changes were found in *Nphs1* or *Podxl* gene expression, encoding nephrin and podoxalyxin, respectively (Figure 5).

### 3.3 Colitis leads to changes in the kallikrein‐kinin system

**FIGURE 6:**
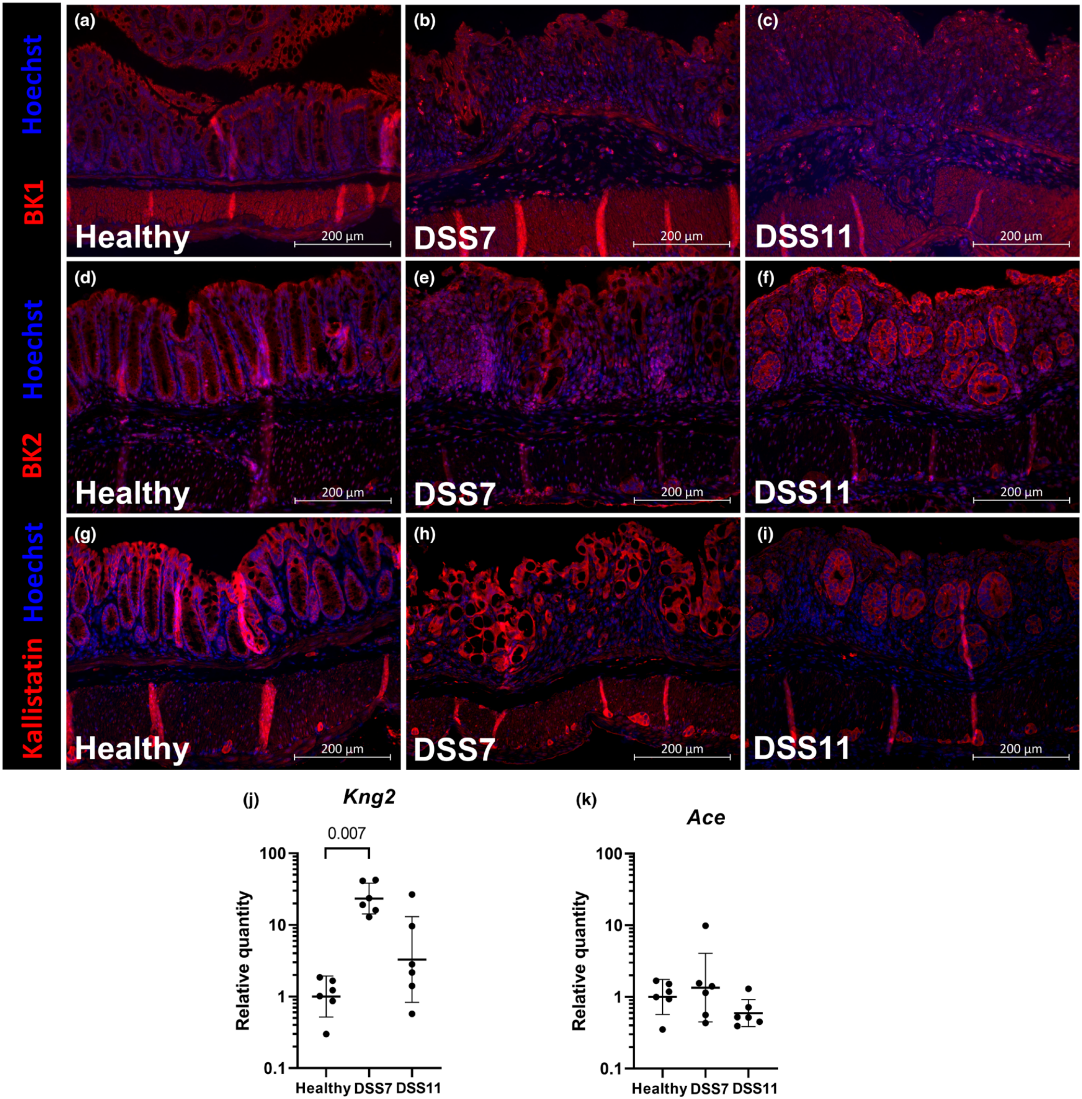
Activation of kallikrein‐kinin system in colitis. Immunofluorescence stainings of colonic BK1 (a–c), BK2 (d–f), and kallistatin (g–i). mRNA expression of *Kng2* and *Ace* in colon measured with RT‐qPCR (j, k). Data are presented with geometric means ± geometric standard deviations. *Ace* = angiotensin‐converting enzyme, BK1 = Bradykinin receptor 1, BK2 = Bradykinin receptor 2, DSS = Dextran sodium sulfate, *Kng2* = kininogen 2.

Activation of intestinal KKS has been implicated in experimental colitis and IBD. We performed immunofluorescence staining of the inducible and constitutively expressed bradykinin receptors BK1 and BK2, respectively, as well as the endogenous inhibitor of kallikrein, kallistatin. In the colon, the basal positive BK1 signal localized to the muscularis and epithelium, but in both groups of colitis mice, cells with strong positive staining were scattered in the damaged mucosa and submucosa (Figure 6a–c). BK2 was highly expressed in epithelial cells, but also in smooth muscle, and myenteric plexus (Figure 6d–f). Kallistatin staining was strongly positive in surface and crypt epithelium and myenteric plexus (Figure 6g–i). The expression pattern of both BK2 and kallistatin reflected the histopathological changes in the crypt epithelium and disappearance of epithelial cells in colitis. BK2 and kallistatin signal were weakly positive in cells of the *lamina propria*. Colonic transcription of *Kng2*, which encodes for kinin precursors low‐ and high‐molecular weight kininogens, and a precursor to bradykinin and kallidin, was increased by colitis and the expression was particularly high at the end of the DSS administration in the DSS7 group (Figure 6j). We also measured the mRNA expression of *Ace*, encoding angiotensin‐converting enzyme, which cleaves and inactivates kinin peptides. Its expression had not changed in the colitis groups (Figure 6k).

**FIGURE 7:**
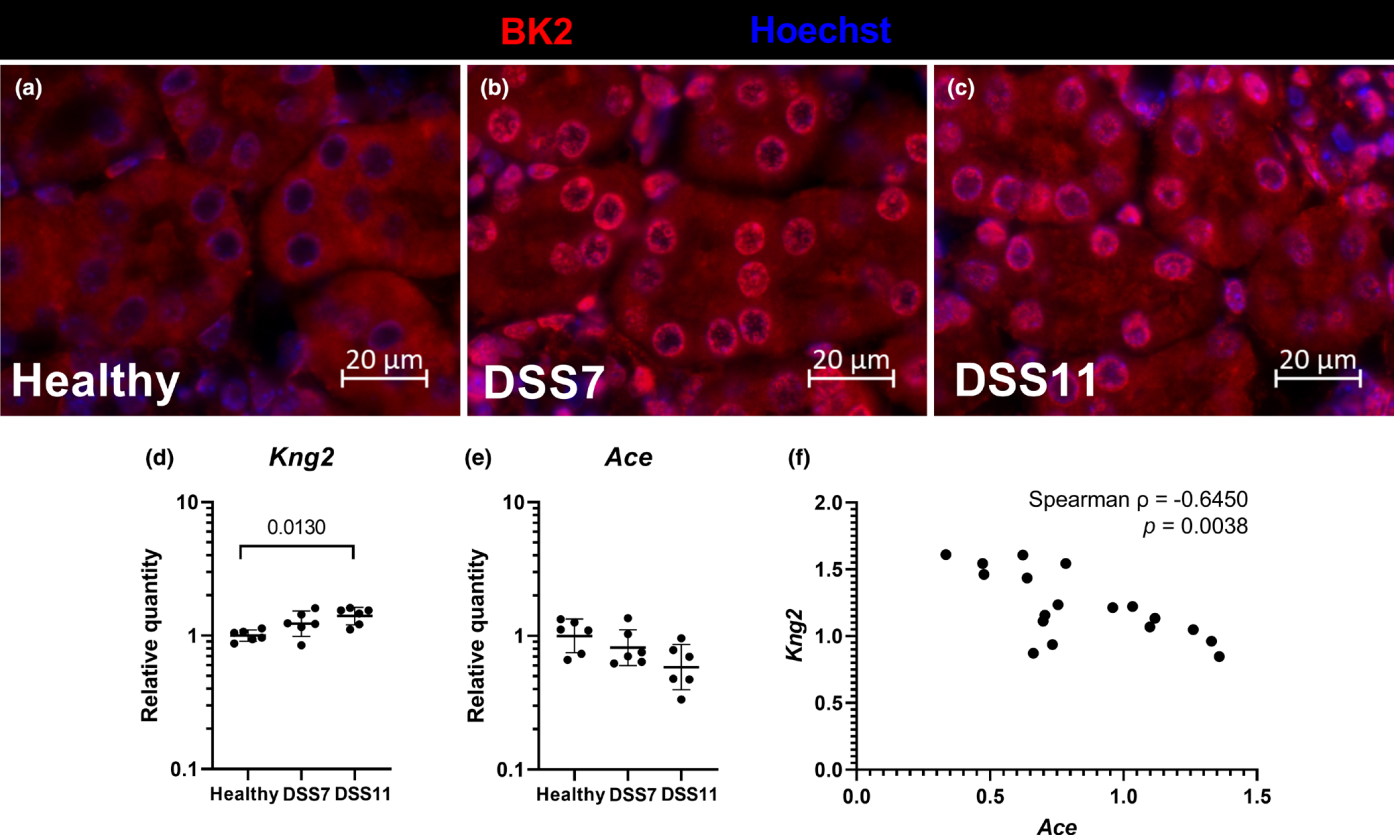
Colitis leads to relocalization of BK2 to nuclear envelope in kidney tubular epithelial cells. Immunofluorescence staining of kidney BK2 (a–c) shown in red. Nuclei in blue. RT‐qPCR analysis of *Kng2* (d) and *Ace* (e). Spearman correlation of kidney *Kng2* and *Ace* mRNA expression (f). Data are presented with geometric means ± geometric standard deviations. *Ace* = angiotensin‐converting enzyme, BK2 = Bradykinin receptor 2, DSS = Dextran sodium sulfate, *Kng2* = kininogen 2.

In the kidneys, the constitutively expressed BK2 was uniformly expressed in the tubular epithelial cells and in some glomerular cells of healthy mice (Figure 7a). Colitis led to a clear increase in BK2 staining around the nuclear envelope in both DSS groups (Figure 7b,c). After the DSS treatment, kidney *Kng2* transcription showed a rising trend in the DSS7 mice and was slightly but significantly elevated in the DSS11 group compared to healthy controls (Figure 7d). *Kng2* transcription correlated negatively with kidney *Ace* transcription (Spearman rho −0.645, *p* = 0.004, Figure 7e,f), which encodes the kinin‐inactivating enzyme, angiotensin‐converting enzyme. Unlike in the colon, the kidney BK1 protein expression was negligible in all groups (data not shown).

### 3.4 Markers of kidney injury correlate positively with colitis severity

**FIGURE 8:**
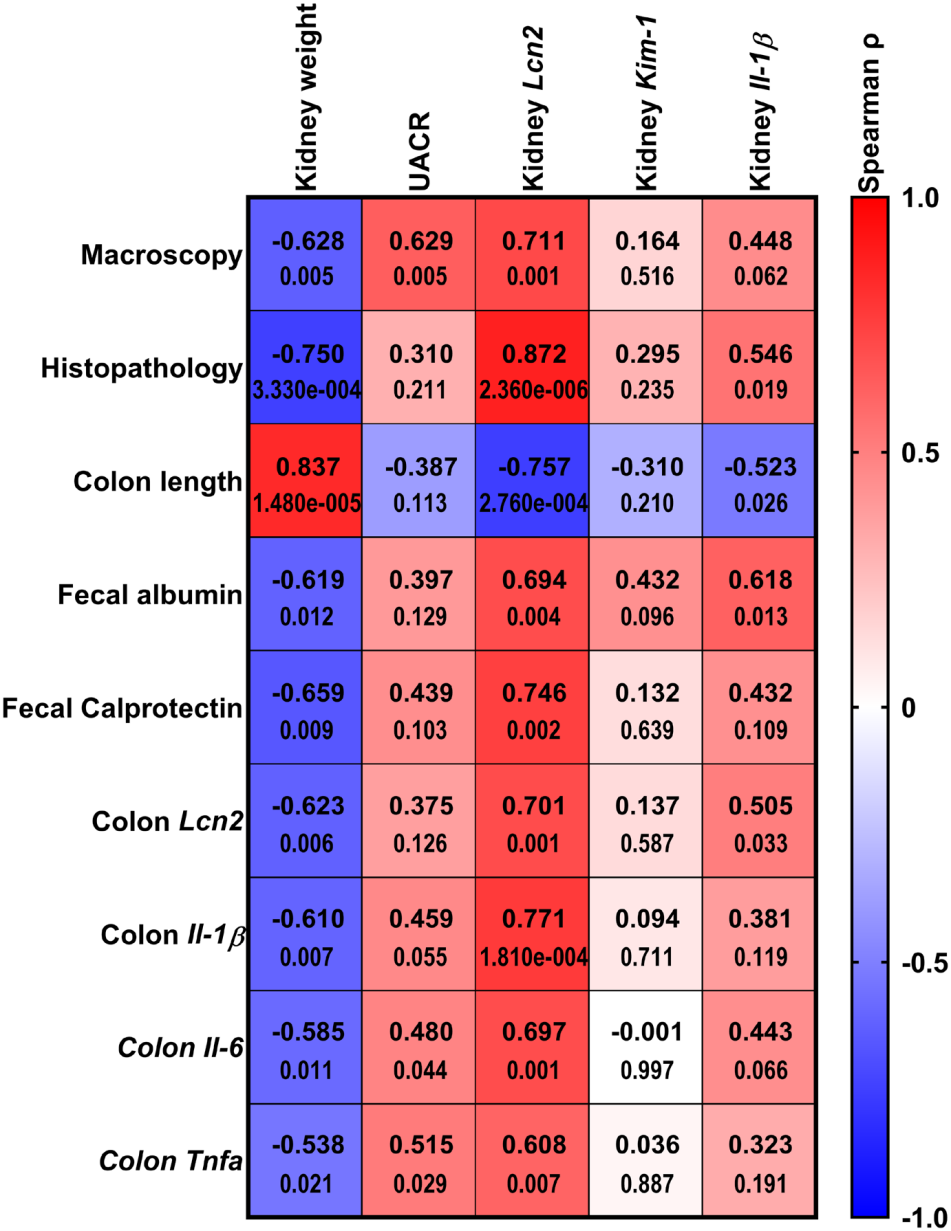
Markers of kidney injury correlate with the severity of colitis. The main colitis parameters (rows): Macroscopic and histopathology scores, colon length, fecal albumin and calprotectin strongly correlate with kidney injury markers (columns); kidney weight, urinary albumin‐to‐creatinine ratio, and kidney *Lcn2* expression. Analysis includes all mice (*n* = 18). The heatmap displays Spearman correlations on top, and *p‐*values below for each pair of correlation. Positive correlations are shown in red and negative correlations in blue.

As the colitis progression in the DSS model can be variable, we calculated correlation coefficients between the kidney injury markers and the markers of colitis in all the mice. There was a correlation between the kidney injury markers; UACR and kidney *Lcn2* expression, and colonic inflammatory markers. Kidney weight and kidney *Il‐1β* correlated with the main markers of colitis, e.g., and histopathological scores, colon length, fecal albumin (Figure 8). The strongest positive correlations were seen between the histopathological scoring of the colitis and the kidney *Lcn2* transcription, macroscopic scoring of the colitis and raw urine albumin, colon length and kidney weight, as well as colonic *Il‐1β* transcription and albuminuria or kidney *Lcn2* transcription. The strongest negative correlations were observed between the histopathological scoring of the colitis and kidney weight, as well as between the colon length and the kidney *Lcn2* transcription.

## 4 DISCUSSION

We investigated the impact of colitis on the kidney phenotype in DSS‐treated mice. Colitis was assessed by a comprehensive set of markers, which demonstrated upregulation of typical injury and inflammation markers, as well as LPS‐detoxifying enzymes in the colon. The severity of the colitis correlated with the appearance of kidney injury markers including albuminuria, and increased mRNA expression of *Lcn2* and *Kim‐1*. Mice with colitis exhibited increased expression of inflammatory markers, *Il‐1β*, c‐Jun, and NF‐κB p65 in their kidneys. We found evidence of activation of both colonic and kidney kallikrein‐kinin systems in DSS colitis. Overall, kidney injury markers correlated strongly with the intestinal damage markers, confirming the connection between intestinal damage and kidney injury.

We assayed a panel of kidney biomarkers that have been linked to various etiologies of human or experimental kidney disease. Several of these markers, including albuminuria, and an increase in kidney *Lcn2* and *Kim‐1* mRNA and c‐Jun protein expression, were altered in our model. Albuminuria is one of the most important markers of kidney disease, and it is often reported as albumin‐to‐creatinine ratio (UACR). We report increased albuminuria following colitis combined with other well‐characterized kidney‐injury markers indicating kidney dysfunction in these mice. Lipocalin 2/neutrophil gelatinase‐associated lipocalin (Lcn2/NGAL) and KIM‐1 are markers of acute kidney injury. Lcn2 is a highly sensitive, but unspecific, tissue damage marker, whereas Kim‐1 expression is the highest in the kidney proximal tubules and they have been found to be increased in different types of human kidney disease (Han et al., 2002; Panduru et al., 2015; Schrezenmeier et al., 2017). These markers are cleaved or leaked into the urine and have been proposed as diagnostic tools to detect early kidney injury (Han et al., 2002; Singer et al., 2013). Kidney expression of Lcn2 in injured kidneys originates mainly from the distal tubules and is upregulated particularly in response to toxic or ischemic injuries (Schrezenmeier et al., 2017), while Kim‐1 is upregulated especially during ischemic acute tubular necrosis, and to a lesser degree in other kidney diseases (Han et al., 2002). The prominent elevation of kidney *Lcn2* mRNA expression in colitis mice at both study timepoints emphasize its sensitivity in detecting early tubular injury, whereas *Kim‐1* was only elevated at the later timepoint consistent with its dependency in disease severity.

Inflammation is well‐established in many types of kidney damage and here, several of the kidney markers altered by colitis, i.e., *Il‐1β*, c‐Jun, and NF‐κB p65, are related to inflammation. This supports the hypothesis that either increased translocation of bacteria directly, or immune cells and proinflammatory mediators activated in the gut can damage the kidneys. Moreover, although the expression of some colitis markers peaked after at the end of the DSS administration, the kidney injury and the inflammation progressed after withdrawal of DSS. Specifically, the expression pattern of c‐Jun changed from focal on day 7 to uniform on day 11, and *Kim‐1* and *Il‐1β* transcription were elevated only at the later time point at day 11, indicating progression of the kidney injury and inflammation secondary to colitis and gut‐barrier dysfunction. The correlation of albuminuria, *Lcn2*, and *Il‐1β* expression with colitis markers strengthens the implication of causality between a gut‐barrier defect and kidney inflammation in this model. c‐Jun forms homodimers or a heterodimer with c‐Fos to form the AP‐1 transcription factor, which regulates genes involved in inflammation, fibrosis, proliferation, and apoptosis (Meng & Xia, 2011). c‐Jun activation has been described in many types of human kidney diseases, including minimal change disease without histopathological damage (De Borst et al., 2007). Likewise, the kidneys of colitis mice in our study were histologically normal, despite the observed albuminuria and elevation of injury markers. These findings suggest a role for nuclear c‐Jun as an early and sensitive marker of kidney damage. Colitis also induced NF‐κB p65 expression in kidney tubules. Many proinflammatory mediators promote NF‐κB and AP‐1 protein activation and nuclear localization. On the other hand, proteinuria itself has been shown to activate NK‐κB in kidney cells (Mezzano et al., 2001).

Earlier studies have shown that mice with DSS and 2,4,6‐trinitrobenzene sulfonic acid (TNBS)‐induced colitis exhibit signs of kidney injury (Chang et al., 2019; Ranganathan, Jayakumar, Manicassamy, & Ramesh, 2013; Ranganathan, Jayakumar, Santhakumar, & Ramesh, 2013; Sui et al., 2024). Ranganathan et al. found increased serum creatinine, myeloperoxidase activity, proinflammatory cytokine expression and dilatation of tubules in kidney tissue following DSS colitis (Ranganathan, Jayakumar, Manicassamy, & Ramesh, 2013; Ranganathan, Jayakumar, Santhakumar, & Ramesh, 2013), while Chang and colleagues reported aberrations in the expression of several critical podocyte proteins, synaptopodin, nephrin, and podocalyxin, as well as different collagens around glomeruli (Chang et al., 2019). Although we could not corroborate all these specific findings, we found clear evidence of kidney inflammation in our DSS model, which further validates the connection between colitis and kidney injury. The phenotypic differences between mouse studies might be explained by different genetic backgrounds, as the choice of mouse strain potentially affects how the kidney injury is manifested. All earlier studies used C57BL/6 mice, whereas BALB/c mice were used in our model. C57BL/6 mice are the most often used strain in colitis studies, and based on the literature and our experience, are more susceptible to DSS colitis than BALB/c mice (Chassaing et al., 2014; Melgar et al., 2005). On the other hand, BALB/c mice are more sensitive than C57BL/6 mice to develop systemic inflammation following influx of gut bacteria (Watanabe et al., 2004), which is why we chose BALB/c for our experiments.

The kallikrein‐kinin system is involved in inflammation and interacts with the contact activation pathway and the renin‐angiotensin system. *Kng2* shows the highest expression in the liver and the kidneys, but our study showed a particularly strong induction of *Kng2* in the colon at the end of the DSS administration in the DSS7 group. On the contrary, kidney expression of *Kng2* increased over time, being the highest at day 11. In the inflamed colon, a strong BK1 signal localized in cells we postulate being mesenchymal cells scattered in mucosa and submucosa. Our results are partly in line with findings in IBD (Stadnicki et al., 2005), of increased BK1 expression in inflamed tissue, although in DSS mice the increased expression did not localize to the enterocytes. Previous animal studies have shown that various types of chemical colitis models, including DSS, TNBS, and indomethacin models, lead to activation of the kallikrein‐kinin system and bradykinin production in the gut and systemically (Hara et al., 2008; Stadnicki et al., 1998; Wang et al., 2018). Knockout of kallikrein‐kinin system components, kininogen, kallikrein, or both bradykinin receptors, was protective against DSS‐ and TNBS‐induced colitis (Wang et al., 2018). However, studies of only BK1 knockout or pharmacological blockade have yielded controversial results regarding their benefit in inhibiting colitis and showed a compensatory increase of BK2 (Hara et al., 2008; Marcon et al., 2013). Moreover, C1‐INH, an inhibitor of kallikrein, treatment has been shown to ameliorate DSS‐induced colitis in mice (Lu et al., 2010). Intestinal kallikrein‐kinin system activation has been reported in IBD (Lehto & Groop, 2018). For example, the endogenous kallikrein inhibitor, kallistatin, was reduced in the crypt epithelium and increased in the *lamina propria* in biopsies from individuals with IBD (Devani et al., 2005). In our study, the kallistatin signal remained strongly positive in enterocytes with only a weak positive signal in *lamina propria*.

We found the colitis to lead to a clear increase and localization of BK2 around the nuclear envelope in the kidney tubular epithelial cells. BK2 activity has been found to be protective in kidney ischemia–reperfusion injury and diabetic kidney disease in mice and rats (Kakoki et al., 2007, 2010). Therefore, activation of the KKS seems to be detrimental in colitis but protective in the kidneys (Kakoki et al., 2007; Wang et al., 2018). Nuclear membrane BK2 was shown to control nuclear signaling via Akt and gene transcription of inducible nitric oxide synthase, iNOS, in rat hepatocytes (Savard et al., 2008). Nuclear Akt activation represses apoptosis (Martelli et al., 1823), which might explain the protective role of KKS activation in the kidneys, and the increase of nuclear membrane BK2 following colitis in our study might be compensatory to the developing kidney injury. Interestingly, although histopathological lesions in the colon were accompanied by a clear increase in BK1 positive cells, the kidney tissue was completely void of any BK1 signal. More studies are needed to explain the apparent discrepant role of BK2 in the gut and the kidneys.

The previous studies regarding the relationship between experimental colitis and kidney injury, and the present one, were all done using the chemical DSS model of colitis. Most of the ingested DSS is excreted in the feces, but some of it is absorbed and eventually excreted in the urine (Kitajima et al., 1999). A direct effect of DSS on the kidneys cannot therefore be completely ruled out. However, a recent study showed that increased bacterial translocation and subsequent increase in proinflammatory mediators worsened the colitis‐induced kidney injury in the DSS model (Manoharan et al., 2022), speaking against a direct effect of DSS on the kidneys. Nevertheless, other, preferably genetic, models of gut barrier injury should be used to confirm the mechanistic relationship between chronic gut‐derived bacterial translocation and kidney injury. A limitation in our model is that some mice in the DSS11 group developed a more severe colitis than expected leading to animals being sacrificed at slightly different time points. It is typical that the DSS model causes colitis of varying intensity (Chassaing et al., 2014). We aimed to investigate how the progression of the colitis affects the kidneys in moderate (DSS7) and advanced (DSS11) stages, and the data demonstrate that progression and increased severity of the colitis leads to more prominent kidney alterations. Chemical models like the DSS model in their acuteness might progress to septic kidney complications at advanced stages. Acute kidney injury is a serious complication of sepsis, but it is not directly comparable to the etiology of kidney complications in human IBD or diabetes (Kumar et al., 2022; Zhang et al., 2018). Yet, the underlying causes are overlapping, i.e., bacterial translocation, increased influx of other bacterial products, and cellular interactions between activated immune cells and target tissues, which makes experimental colitis models important for studying diagnostics and treatment of gut barrier defect‐induced kidney injury.

In conclusion, disruption of the gut barrier by DSS colitis leads to kidney inflammation and signs of early kidney injury. Of importance, the degree of kidney injury correlates with the severity of colitis. The acute colitis led to an increase in markers relevant to human kidney diseases, including albuminuria and markers of inflammation without visible histopathological damage. We found evidence of tissue KKS activation and changes in bradykinin receptor expression patterns, whose role in the pathogenesis of human gut and kidney related disorders remains to be elucidated in future studies.

## AUTHOR CONTRIBUTIONS

Conception and design: H. S., M. L., S. L. Data acquisition: H. S., K. A., A. P., J. L. Analysis: H. S. Interpretation of data: H. S., M. L., S. L., J. L., N. S., P‐H. G. Drafting the manuscript: H. S., M. L. All authors revised and approved the final version of the manuscript.

## FUNDING INFORMATION

This work was funded by Folkhälsan Research Foundation, Wilhelm and Else Stockmann Foundation, Liv och Hälsa Society, Helsinki University Hospital Research Funds, Päivikki and Sakari Sohlberg Foundation, Diabetes Research Foundation, Novo Nordisk Foundation (#NNF OC0013659, NNF23OC0082732), Medical Society of Finland (Finska Läkaresällskapet) Einar and Karin Ström Foundation. The open access was funded by the Helsinki University Library.

## CONFLICT OF INTEREST STATEMENT

P‐H.G. has received lecture fees from Astellas Pharma, AstraZeneca, Bayer, Berlin Chemie, Boehringer Ingelheim, Eli Lilly, Elo Water, Genzyme, Medscape, Menarini, Merck, Sharp & Dohme, Mundipharma, Novartis, Novo Nordisk, PeerVoice, Sanofi, and Sciarc. P‐H. G reports being an advisory board member for AbbVie, Astellas Pharma, AstraZeneca, Bayer, Boehringer Ingelheim, Eli Lilly, Janssen Pharmaceuticals, Medscape, Merck, Sharp & Dohme, Mundipharma, Nestlé, Novartis, Novo Nordisk, and Sanofi. The other authors have no conflicts of interest to declare.

## ETHICS STATEMENT

The animal experiments were authorized by the Finnish Project Authorization Board under the permit ESAVI/6504/2020.

## Data Availability

The data are available from the corresponding author upon a reasonable request.
